# Continuous Spinal Anesthesia in Frail Patients Undergoing Orthopedic Hip and Knee Revision Surgery: Advantages, Indications, and Risk Management—A Single-Center Retrospective Experience

**DOI:** 10.3390/jcm15083174

**Published:** 2026-04-21

**Authors:** Yazan Abu Salem, Emilia Cialdella, Vincenzo Simili, Federica Martorelli, Giuseppe Monteleone, Francesco Tasso, Berardo Di Matteo, Giuseppe Anzillotti, Elizaveta Kon, Marco Scardino

**Affiliations:** 1 IRCCS Humanitas Research Hospital, Via Manzoni 56, 20089 Rozzano, Italy; 2 Department of Biomedical Sciences, Humanitas University, Via Rita Levi Montalcini 4, 20072 Pieve Emanuele, Italy

**Keywords:** continuous spinal anesthesia (CSA), frail patient, revision arthroplasty, hemodynamic stability, hip and knee revision, perioperative management, high-risk anesthesia

## Abstract

**Background**: Frail patients undergoing hip and knee revision surgery represent a major anesthetic challenge because of advanced age and multiple comorbidities. Continuous spinal anesthesia (CSA) with titrated low-dose levobupivacaine may offer a potentially useful alternative to general anesthesia or single-shot spinal anesthesia in this high-risk population. **Methods**: A retrospective review was conducted of ASA II-III patients who underwent complex hip and knee revision surgeries between February and October 2024 under CSA. The technique was performed using a 25-gauge spinal catheter with incremental boluses of 0.25% levobupivacaine (2.5 mg). Hemodynamic parameters, including mean arterial pressure (MAP), stroke volume index (SVI), and cardiac index (CI), were continuously monitored using the EV1000 hemodynamic monitoring system. Postoperative complications were recorded. **Results**: 37 high-risk patients were included in the study. Catheter placement was successful in all patients, with no conversions to general anesthesia. MAP decreased by a mean of 14.6% after boluses (*p* < 0.05); 9 patients (24.3%) experienced reductions ≥ 20%, but all remained >65 mmHg and responded to fluid therapy. CI and SVI decreased by 10.1% and 10.5%, respectively (*p* < 0.05), without clinical instability. No major complications (neurological injury, infection, post-dural puncture headache) were observed. **Conclusions**: In this retrospective single-center experience, CSA with titrated low-dose levobupivacaine was feasible and associated with stable hemodynamic profiles and a low rate of complications in frail patients undergoing complex lower-limb revision surgery. However, given the absence of a control group and the limited sample size, these findings should be interpreted cautiously. Further prospective comparative studies are needed to better define the role of CSA in high-risk orthopedic patients.

## 1. Introduction

Complex knee and hip arthroplasty revision procedures in patients with increased frailty represent a major challenge in orthopedic surgery. Frailty is a multidimensional clinical syndrome reflecting decreased physiological reserve and increased vulnerability to stressors. Both the technical complexity of the surgery and the fragile clinical status of these patients increase perioperative risk [1,2]. With population aging, the demand for revision arthroplasty is steadily rising, particularly in patients with complications such as infection, thromboembolism, dislocation, or prosthetic wear [3,4]. In this context, anesthetic management plays a pivotal role in influencing outcomes.

General anesthesia and single-shot spinal anesthesia, despite being widely used, suffer from limitations, such as cardiovascular and respiratory depression, and limited duration with reduced flexibility [5,6].

In contrast, continuous spinal anesthesia offers numerous advantages, such as excellent hemodynamic stability, reduced incidence of cardiovascular and respiratory complications, absence of surgical time limitations, as well as improved postoperative pain control [6,7].

Continuous spinal anesthesia (CSA) allows fir controlled administration of low-concentration anesthetics, which not only reduces the risk of systemic side effects but also permits faster recovery and early mobilization [8]. Recent studies have reported that CSA improves outcomes in elderly and frail patients undergoing major orthopedic surgery, including reduced hemodynamic instability and perioperative complications [7,9,10]. However, data on its use in complex revision procedures remain limited.

The aim of this single-center retrospective study is to evaluate the safety and efficacy of CSA using low-dose 0.25% levobupivacaine in patients with increased frailty and high anesthetic risk undergoing hip and knee revision surgery.

## 2. Materials and Methods

### 2.1. Patient Selection

This retrospective single-center study included consecutive patients who underwent continuous spinal anesthesia (CSA) between February and October 2024. Patient selection was guided by procedural complexity (revision hip or knee arthroplasty), anticipated surgical duration, bleeding risk, and expected hemodynamic impact. Frailty was not formally assessed using validated instruments such as the Clinical Frailty Scale [11] or the Fried frailty phenotype [12]. Instead, in this retrospective analysis, frailty was pragmatically defined based on a combination of advanced age, ASA classification, and the presence of multiple comorbidities (e.g., cardiovascular disease, COPD, diabetes, and obesity). This pragmatic definition was adopted to reflect real-world clinical practice, where anesthetic risk stratification is often based on comprehensive clinical judgment rather than standardized frailty scoring systems. Particular attention was given to patients with comorbidities that could compromise perioperative stability. Patients classified as American Society of Anesthesiologists (ASA) III–IV were considered high-risk.

The revision procedures included complex hip and knee arthroplasties performed for common indications such as prosthetic loosening, periprosthetic joint infection, instability, and implant wear. Although detailed classification of revision types (e.g., one-stage vs. two-stage revision, component exchange vs. full revision) was not systematically recorded in this retrospective dataset, all procedures were considered technically demanding and associated with prolonged operative time and potential for significant blood loss.

This study was conducted in accordance with the ethical standards of the institutional research committee and the Declaration of Helsinki, and all participants provided written informed consent [13]. In cases where patients were receiving prophylactic low-molecular-weight heparin (LMWH), the spinal catheter was inserted at least 12 h after the last LMWH administration, in accordance with current guidelines [14,15].

Exclusion criteria included: (1) coagulation disorders (platelet count < 100,000/mm^3^, international normalized ratio (INR) > 1.5, activated partial thromboplastin time (aPTT) > 40 s); (2) ongoing anticoagulant or antiplatelet therapy not discontinued appropriately; (3) pre-existing neurological deficits (e.g., cauda equina syndrome, myelopathy); (4) systemic or local infections; (5) allergy to amide local anesthetics; (6) significant vertebral abnormalities; (7) hemodynamic instability (persistent hypotension, shock); (8) severe cardiac disease (e.g., unstable coronary artery disease).

### 2.2. Anesthetic Protocol

**Figure 1:**
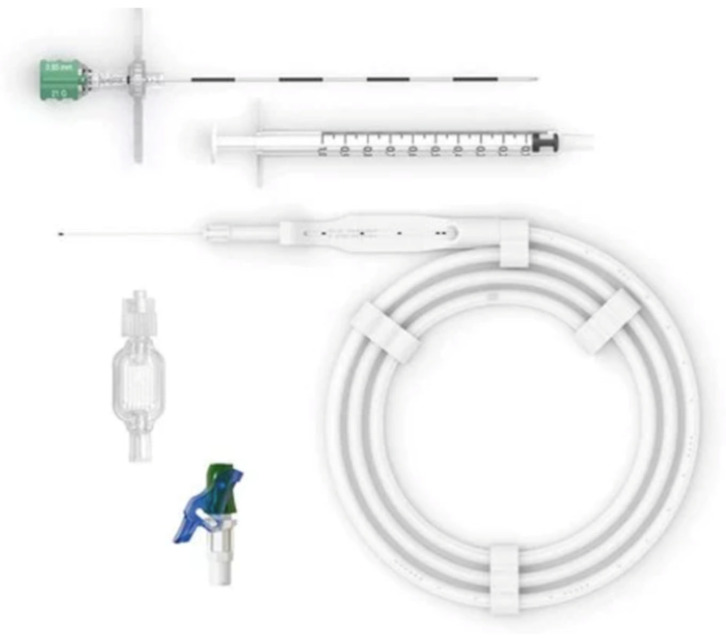
IntraLong^®^ set for continuous spinal anesthesia (PAJUNK^®^ GmbH, Geisingen, Germany). IntraLong^®^ is provided by PAJUNK^®^ in dedicated sets containing the following components: SPROTTE^®^ SPECIAL tip needle with fixation plate, 25–27 G intrathecal catheter with fully radiopaque stylet in protective container, Clamping adapter, Terminal cap, 0.2 μm antibacterial filter, Graduated syringe, Luer-lock connector.

CSA was performed using the Pajunk IntraLong set (Pajunk GmbH, Geisingen, Germany) (Figure 1). In the preoperative area, baseline vital parameters were recorded, intravenous (IV) access was secured (16 G or 18 G cannula), and patients were prehydrated with crystalloids (5 mL/kg over 20–30 min). Sedation was achieved with IV midazolam (2–4 mg), titrated to patient comfort and agitation level.

**Figure 2:**
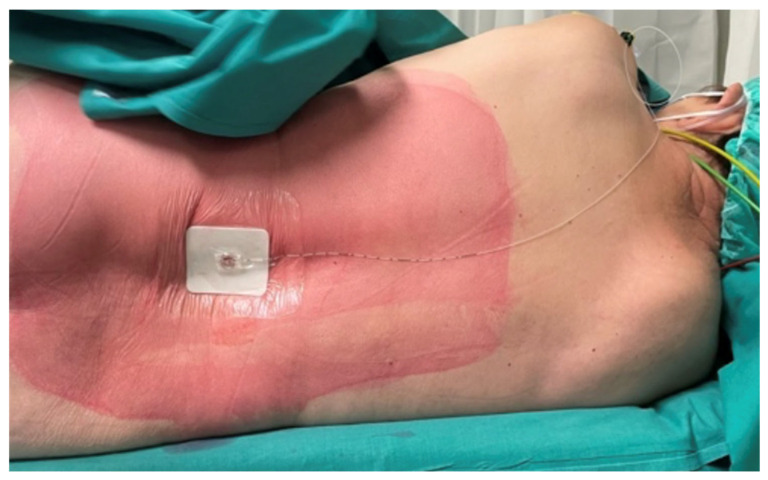
Positioning of the spinal catheter in continuous spinal anesthesia. Lateral view of the patient in lateral decubitus position with intrathecal catheter placed for CSA. The area is properly disinfected and the catheter fixed with transparent sterile dressing.

After careful skin disinfection and sterile field preparation, anesthesia was performed with the patient in sitting or lateral decubitus position, using a median approach at the L2–L3 or L3–L4 interspaces (Figure 2). After identifying the interspace, local infiltration with 2% lidocaine was performed. A 21 G Sprotte needle was advanced until the subarachnoid space was reached, confirmed by the outflow of cerebrospinal fluid (CSF). The skin-to-subarachnoid space distance was measured to ensure correct catheter placement.

A 25 G spinal catheter was then advanced 3–4 cm cranially into the subarachnoid space. After needle removal, the catheter was connected to a locking adapter and tested by aspirating cerebrospinal fluid with a 1 mL syringe, confirming proper placement. Once verified, the catheter was connected to an antibacterial filter pre-filled with 0.25% levobupivacaine (about 1 mL) and secured with transparent dressing.

An initial bolus of 2–3 mL of 0.25% levobupivacaine (5–7.5 mg) was administered, followed by incremental boluses of 1 mL (2.5 mg) as required. Levobupivacaine was chosen over racemic bupivacaine due to its lower cardiotoxicity and neurotoxicity, attributed to the S-enantiomer’s reduced affinity for cardiac and neuronal ion channels [16].

### 2.3. Clinical Assessment

Patients were continuously monitored with a standard monitoring, including three-lead electrocardiography (ECG), heart rate, pulse oximetry and non-invasive blood pressure (NIBP).

Stroke Volume Index (SVI) and Cardiac Index (CI) were continuously monitored using the EV1000 hemodynamic monitoring system, while mean arterial pressure (MAP) was recorded every 5 min during surgery. Changes in SVI, CI, and MAP before and after 2.5 mg bolus of 0.25% levobupivacaine were analyzed. Hypotension was defined as a decrease ≥ 20% from baseline [17].

Urine output was measured with urinary catheterization, and blood loss was quantified, with reinfusion of cell-saver blood when possible or transfusion of red blood cells (RBCs) and plasma in major bleeding (>1000 mL). Patients were equipped with anti-decubitus devices, warming systems (forced hot air, thermal blanket), and DVT prophylaxis stockings on the contralateral limb.

### 2.4. Anesthetic Titration

Anesthesia was titrated with incremental boluses of 1 mL (2.5 mg) of 0.25% levobupivacaine, tailored to surgical requirements (e.g., muscle relaxation) and patient comfort.

### 2.5. Intraoperative and Postoperative Management

Intraoperatively, patients received: tranexamic acid (1 g), dexamethasone (8 mg), ondansetron (4 mg), metoclopramide (10 mg), and omeprazole (40 mg) to prevent bleeding and reduce postoperative nausea and vomiting (PONV). At the end of the procedure, the spinal catheter was removed, ensuring tip integrity with the black marker visible.

Patients were monitored in the recovery room for at least 90 min, and adverse effects (nausea, vomiting, bradycardia, or hypotension) were recorded. Low-molecular-weight heparin (LMWH) or non-vitamin K antagonist oral anticoagulant (NOAC) prophylaxis was resumed 8–12 h after catheter removal, following international recommendations [14,15].

### 2.6. Follow-Up and Complications

Patients were evaluated in the ward for complications such as peripheral nerve root injury (radiculopathy, lumbar pain, cauda equina syndrome); central nervous system complications (meningitis, spinal abscess, spinal hematoma) and post-dural puncture headache (PDPH).

## 3. Results

A total of 37 patients underwent continuous spinal anesthesia with 0.25% levobupivacaine titrated in 1 mL (2.5 mg) boluses. Most were female (*n* = 27, 73%), and the median age was 70 years (range 55–77).

The majority of patients (32, 86.5%) underwent hip revision surgery, while 4 patients (10.8%) had knee revision surgery. In one case, a patient with achondroplasia (dwarfism) underwent primary hip arthroplasty. Despite being a primary procedure, this case was included because of the markedly increased anatomical and anesthetic complexity, which made it comparable to revision surgery in terms of surgical difficulty, duration, and perioperative risk. All procedures were classified as major revision surgeries, characterized by prolonged operative time and expected increased intraoperative complexity compared to primary arthroplasty. Catheter placement was successful in all cases, with 27 patients (73%) at L3–L4 and 10 patients (27%) at L2–L3. Most patients (33, 89.2%) were positioned laterally, while 4 (10.8%) were seated. Initial anesthetic doses were 5 mg or 7.5 mg of levobupivacaine. Mean surgery duration was 169.5 min, with effective anesthesia management via incremental boluses of 1 mL (2.5 mg) of levobupivacaine 0.25%. Although intraoperative blood loss was routinely monitored and managed according to institutional protocols, detailed quantitative data were not consistently available for all patients and therefore were not included in the analysis. The mean interval between boluses was 84 min. Comorbidities were common in this cohort: hypertension was present in 37.8% of patients, while 29.7% had additional conditions including chronic obstructive pulmonary disease (COPD), diabetes mellitus, obesity, or valvular heart disease. Demographic characteristics of the included patients are listed in Table 1.

### 3.1. Hemodynamic Changes

**Figure 3:**
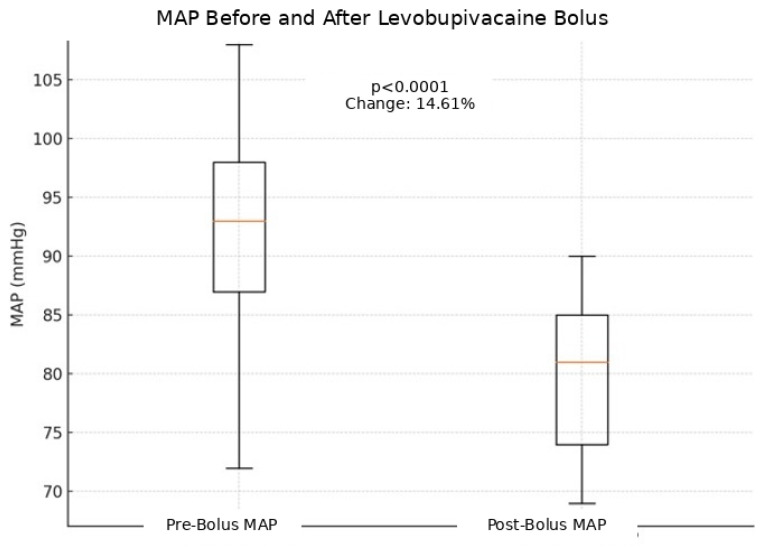
Variation in MAP values before and after bolus of Levobupivacaine 0.25%, with an average reduction of 14.61%.

**Figure 4:**
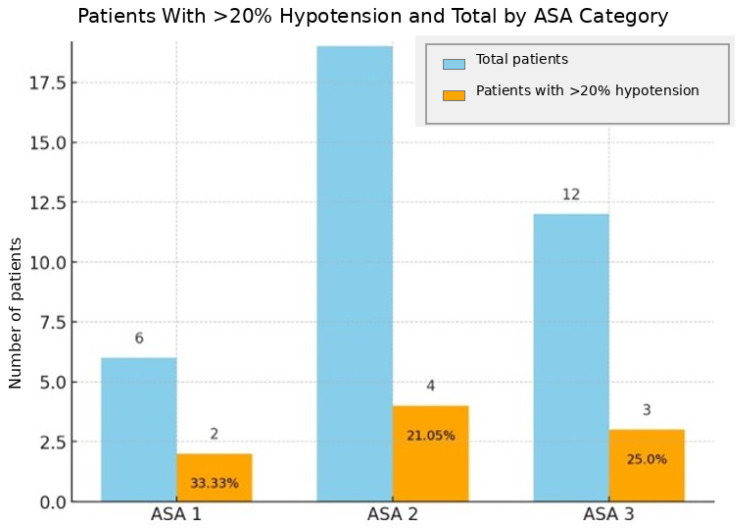
Distribution of patients with hypotension ≥ 20% by ASA class. Percentages: ASA 1 (33.33%), ASA 2 (21.05%), ASA 3 (25%).

**Figure 5:**
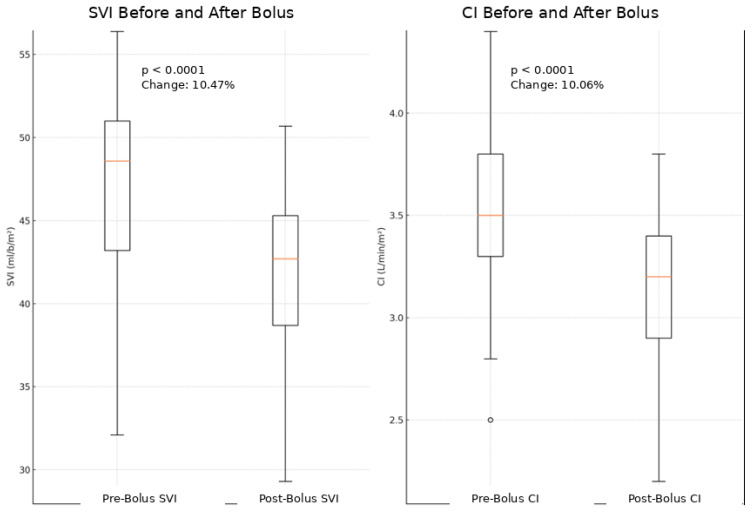
Variation in SVI and CI before and after bolus of Levobupivacaine 0.25%. Significant reduction in mean Stroke Volume Index values (−10.47%) and Cardiac Index (−10.06%) after administration.

After levobupivacaine boluses, mean arterial pressure (MAP) decreased by 14.6% on average (*p* < 0.05) (Figure 3). Nine patients (24.3%) experienced MAP reductions ≥ 20%, distributed as follows: ASA I: 2/6 (33.3%), ASA II: 4/19 (21.0%), ASA III: 3/12 (25.0%) (Figure 4). Stroke Volume Index (SVI) and Cardiac Index (CI) decreased by 10.5% and 10.1%, respectively (*p* < 0.05) (Figure 5). All hypotensive episodes were transient and resolved with fluid administration, without the need for vasopressors. Hemodynamic outcomes are listed in Table 2.

### 3.2. Complications

No conversions to general anesthesia occurred. No major neurological, infectious, or hematological complications were observed. In particular, there were no cases of post-dural puncture headache (PDPH), meningitis, spinal abscess, or spinal hematoma.

## 4. Discussion

CSA with 0.25% levobupivacaine proved to be a safe and effective technique for frail patients with significant comorbidities and high perioperative risk. Patients, mostly undergoing hip and knee revisions, presented complex clinical profiles with high prevalence of hypertension, chronic obstructive pulmonary disease (COPD), mitral insufficiency, obesity, and type 2 diabetes. These comorbidities often pose a high risk of hemodynamic instability and increase the risk of perioperative morbidity, under both general and single-shot spinal anesthesia [18,19,20,21,22].

An important aspect to consider when interpreting our findings is the absence of a control group. Therefore, although CSA was associated with stable hemodynamic parameters, absence of conversion to general anesthesia, and no major complications in this cohort, no direct comparison can be made with general anesthesia or single-shot spinal anesthesia. As a result, the present study should be interpreted as a descriptive analysis of the feasibility and perioperative safety profile of CSA in a selected high-risk population, rather than evidence of superiority over other anesthetic techniques.

Continuous spinal anesthesia has been reported in the literature to offer several potential advantages over general anesthesia and single-shot spinal anesthesia, particularly in patients with limited cardiopulmonary reserve [9,23,24]. However, in the absence of a control group, our findings cannot confirm these advantages, but only support the feasibility and safety of CSA in this specific clinical setting. The incremental titration of anesthetic boluses allows individualized adjustment of block intensity and duration, minimizing sudden sympathetic block and hypotension [22,25]. In our study, CSA was associated with maintained intraoperative hemodynamic stability, avoidance of conversion to general anesthesia, and the ability to allow tailored dosing even during long and complex procedures.

Advanced hemodynamic monitoring using the EV1000 system confirmed that CSA ensured precise control of mean arterial pressure (MAP), cardiac index (CI)**,** and stroke volume index (SVI) maintaining stability even in patients with reduced cardiac reserve.

The hemodynamic results obtained in this study support the hypothesis that CSA with titrated levobupivacaine is a safe choice, particularly suited for frail patients [26]. The analysis of mean arterial pressure (MAP) before and after bolus administration showed an average variation of 14.61%, without episodes of clinically relevant hypotension. This finding is consistent with the literature, which highlights that a reduction between 10 and 30% from baseline is physiological after sympathetic block [27,28]. We defined hypotension as a reduction in MAP ≥ 20%, and within our cohort only 9 out of 37 patients (24.32%) showed a significant decrease in MAP, which nevertheless remained > 65 mmHg. In none of these cases was it necessary to administer vasopressors.

These data suggest that CSA, if properly managed with gradual drug titration, allows for the prevention of clinically dangerous hypotensive episodes, making the technique a potentially safe option in this high-risk population [29]. Most cases of MAP reduction were treated with fluid administration, demonstrating that the conservative approach adopted was sufficient to maintain hemodynamic stability.

Moreover, continuous monitoring of parameters such as cardiac index (CI) and stroke volume index (SVI) was essential to assess the impact of continuous spinal anesthesia on cardiac function. The observed variations, with a percentage decrease of 10.1% in CI and 10.5% in SVI before and after bolus administration, although statistically significant (*p* < 0.05), had no relevant clinical repercussions. This suggests that, although anesthesia led to expected physiological changes, these changes did not result in clinical instability or hemodynamic damage.

Additionally, several studies indicate that a reduction in SVI around 10–15% is predictable during subarachnoid anesthesia, particularly following vasodilation induced by sympathetic block. This effect translates into decreased venous return (preload), reduced ventricular filling, and consequently lower stroke volume and cardiac output [30,31]. Regarding fluid management, preventive fluid administration (preload augmentation) is often recommended to reduce the risk of hypotension [32]. However, recent studies suggest that fluid therapy may be more effective when administered reactively, that is, when significant drops in blood pressure or other signs of hypotension are observed [33,34,35].

This hemodynamic stability, even in the presence of significant variations, further supports the safety of CSA in patients with cardiac impairment or low cardiovascular reserve. In the context of major lower limb surgery, where physiological demands and risks of decompensation are high, the use of titrated levobupivacaine was associated with optimal balance between anesthetic efficacy and hemodynamic protection.

Regarding adverse events, no major complications such as post-dural puncture headache (PDPH), meningitis, spinal hematoma, or cauda equina syndrome were observed. This is consistent with contemporary evidence demonstrating low complication rates of CSA when modern fine-gauge intrathecal catheters and standardized management protocols are used [36,37]. Concerns about PDPH and neurological injury have historically limited CSA use, but contemporary evidence suggests that with 25–28 G catheters, complication rates are negligible [37,38].

A recent review by Ye & Selander challenged several “myths” surrounding CSA, highlighting that historical concerns over high PDPH incidence and neurological risks are largely outdated when modern equipment and techniques are employed [39]. Similarly, Elfety et al. demonstrated that continuous spinal anesthesia with titrated dosing via catheter is a safe and effective approach in high-risk elderly patients [40].

Our findings further support that CSA can be performed safely, even in patients receiving antithrombotic prophylaxis, provided guidelines for neuraxial anesthesia and anticoagulation are followed [41].

While most prior CSA studies involve elective arthroplasty in elderly patients, few specifically address revision procedures, which are typically longer and more demanding. Koole et al. demonstrated that continuous spinal anaesthesia provides superior haemodynamic control, with a lower incidence of intraoperative hypotension compared with single-shot spinal or general anaesthesia in patients undergoing hip surgery [7]. Aksoy et al. demonstrated that continuous spinal anaesthesia allows stable haemodynamic conditions with minimal cardiovascular effects, while providing effective intraoperative anaesthesia and sustained postoperative analgesia in high-risk patients undergoing hip fracture surgery [42].

Mady et al. demonstrated that continuous spinal anaesthesia results in lower vasopressor use and reduced anaesthetic requirements compared with single-dose spinal anaesthesia in octogenarians undergoing hip surgery, supporting its role in enhancing haemodynamic stability in this high-risk population [43].

Lux et al., in a study of 1212 patients undergoing lower limb surgery, reported that the use of a 28-gauge microcatheter for CSA did not lead to significant complications [44].

Lairez et al. conducted an echocardiographic study on 49 patients to assess the hemodynamic impact of single-shot spinal anesthesia with low doses of anesthetic (7.5 mg of 0.5% bupivacaine with 5 μg sufentanil), showing a decrease in CI between 10 and 22% compared to baseline, which could be compensated either with fluid administration alone or by using even lower doses of anesthetic through continuous spinal anesthesia, which is exactly what was performed in this study and has already been extensively discussed [45].

Our findings are consistent with and extend these results to revision arthroplasty, suggesting that CSA is equally effective in this setting. Importantly, we employed one of the lowest levobupivacaine dosing strategies reported to date, supporting that safe and adequate anesthesia can be achieved with minimal drug exposure. Similar principles have also been applied outside orthopedics: Castellani et al. reported that CSA reduced morbidity in frail octogenarians undergoing radical cystectomy, further supporting its role in high-risk surgical populations [46].

The results of this study have several implications: (1) CSA as a first-line option: for frail patients with ASA II–III status undergoing complex revision procedures, CSA may represent a safe and effective primary anesthetic strategy; (2) Tailored anesthetic depth: Incremental dosing allows precise adjustment to surgical needs, minimizing risks of over- or under-anesthesia; (3) Hemodynamic protection: The combination of low-dose levobupivacaine and gradual titration was associated with cardiovascular stability, even in patients with limited reserve.

This study has some limitations as well. First, the sample size is relatively small and all cases were performed in a single institution with experienced anesthesiologists, which may limit the generalizability of the findings. Therefore, the results should be considered preliminary and interpreted with caution. Larger, multicenter studies are required to validate these findings across different clinical settings. A major limitation of this study is the absence of a control group (e.g., general anesthesia or single-shot spinal anesthesia), which precludes any direct comparison and limits causal inference. Consequently, it is not possible to determine whether CSA is superior to other anesthetic techniques, and the observed outcomes should be interpreted as descriptive findings within a selected cohort. Comparative or randomized studies are required to establish the relative advantages of CSA. Another limitation of this study is the lack of detailed orthopedic surgical data, including specific types of revision procedures, indications for revision (e.g., infection, aseptic loosening, or periprosthetic fracture), and precise intraoperative blood loss. These variables could have provided a more comprehensive characterization of surgical complexity. However, the primary focus of this study was the anesthetic management and hemodynamic impact of continuous spinal anesthesia in a high-risk population. Another limitation of this study is the lack of a standardized frailty assessment using validated tools such as the Clinical Frailty Scale or the Fried criteria. Frailty was instead defined using a pragmatic approach based on ASA classification and comorbidity burden. Although this reflects common clinical practice in anesthetic risk stratification, it may limit comparability with studies adopting formal frailty indices. Finally, long-term follow-up for late complications (neurological sequelae, chronic pain) was not available. Future prospective comparative studies are warranted to further define the role of CSA in complex revision arthroplasty.

## 5. Conclusions

Continuous spinal anesthesia with low-dose, titrated levobupivacaine was feasible and associated with stable hemodynamics and a low rate of complications in this cohort of frail patients undergoing complex hip and knee revision surgery. However, given the retrospective design, small sample size, and single-center nature of the study, these findings should be considered preliminary and not interpreted as demonstrating superiority over other anesthetic techniques. Larger, multicenter, and prospective studies are needed to confirm these results and better define the role of CSA in high-risk orthopedic patients.

## Figures and Tables

**Table 1:** Patients’ characteristics of the included patients.

Variable	Value
Age, years, median (IQR)	70 (55–77)
Female sex, n (%)	27 (73.0)
ASA II, n (%)	27 (56.8)
ASA III, n (%)	10 (27.0)
Hypertension, n (%)	14 (37.8)
Other comorbidities (COPD, diabetes, obesity, valvular disease)	11 (29.7)
Hip revision arthroplasty, n (%)	32 (86.5)
Knee revision arthroplasty, n (%)	4 (10.8)
Primary hip arthroplasty (dwarfism), n (%)	1 (2.7)
Surgical duration, min, mean (SD)	169.5 (±34.2)
Successful catheter placement, n (%)	37 (100)

**Table 2:** Hemodynamic outcomes during CSA.

Parameter	Mean % Change After Bolus	*p*-Value	Clinical Impact
Mean arterial pressure (MAP)	−14.6%	<0.05	9/37 patients (24.3%) with ≥20% drop, all >65 mmHg
Stroke Volume Index (SVI)	−10.5%	<0.05	No clinical instability
Cardiac Index (CI)	−10.1%	<0.05	No clinical instability
Need for vasopressors	0%	—	All managed with fluids
Conversion to general anesthesia	0%	—	—
Severe complications	0%	—	—

## Data Availability

The original contributions presented in this study are included in the article. Further inquiries can be directed to the corresponding author.
